# Highly Divergent Dengue Virus Type 2 in Traveler Returning from Borneo to Australia

**DOI:** 10.3201/eid2212.160813

**Published:** 2016-12

**Authors:** Wenjun Liu, Paul Pickering, Sebastián Duchêne, Edward C. Holmes, John G. Aaskov

**Affiliations:** Australian Army Malaria Institute, Brisbane, Queensland, Australia (W. Liu, P. Pickering J.G. Aaskov);; University of Sydney, Sydney, New South Wales, Australia (S. Duchene, E.C. Holmes);; Queensland University of Technology, Brisbane (J.G. Aaskov)

**Keywords:** dengue virus, dengue virus type 2, DENV-2, viruses, traveler, divergence, phylogeny, evolution, sylvatic cycle, Borneo, Australia

## Abstract

Dengue virus type 2 was isolated from a tourist who returned from Borneo to Australia. Phylogenetic analysis identified this virus as highly divergent and occupying a basal phylogenetic position relative to all known human and sylvatic dengue virus type 2 strains and the most divergent lineage not assigned to a new serotype.

Outbreaks of dengue have been reported for several centuries in the Old World and the New World. Dengue is a major cause of illness and death globally, and there are high levels of infection in many tropical and subtropical localities populated by *Aedes* mosquito vectors. Despite their role in public health, the origin of the 4 serotypes of dengue virus (DENV-1–DENV-4) that are the causative agents of what now is defined as dengue remains unclear.

In 1967, Rudnick et al. (*1*) combined a fragmentary body of evidence to propose that dengue might have a sylvatic (jungle) cycle similar to that of another flavivirus (yellow fever virus). Isolation of 4 serotypes of DENV from humans in the Asia–Pacific region during 1943–1956 (*2**–**4*) was compatible with the idea that DENV might have originated in this region. Genome sequences of DENV-2 and DENV-4 isolated from sylvatic settings (i.e., from nonhuman primates) occupied basal positions on phylogenetic trees of those serotypes, which suggested that each DENV serotype evolved separately in sylvatic settings before later, independent, cross-species transmission to humans in urban and semiurban settings (*5**,**6*).

DENV-2 and DENV-4 have been isolated from nonhuman primates and occupy divergent phylogenetic positions, which suggests that that they are truly sylvatic. In contrast, no sylvatic strains have been identified as DENV-3, and an early sylvatic strain of DENV-1 probably was a spillback from humans to other primates (*6*). However, a highly divergent sequence of DENV-1, which was isolated from a patient who had vacationed in Brunei, was recently reported (*7*). This virus was basal to all other strains of DENV-1 by phylogenetic analyses, which suggests the presence of another sylvatic virus lineage in Southeast Asia, albeit of unknown animal origin.

Despite the central role of sylvatic viruses in our understanding of evolution and emergence of human dengue (*6*), to our knowledge, there are no reports of continuous transmission of sylvatic strains of DENV in a truly sylvatic setting. Much of the uncertainty over the nature and role of sylvatic DENV arises because of the small number of such isolates available and the lack of studies of DENV ecology outside a human setting. Thus, although phylogenetic divergence alone is insufficient to definitively prove the existence of sylvatic transmission, it at least shows that a greater diversity of viruses exist than are usually assigned as causing dengue in humans. In this report, we describe a highly divergent strain of DENV-2 isolated from an acute-phase serum specimen from a patient (human ethical approval for this study precludes identification of the patient) in whom dengue developed after the patient returned from a vacation in Borneo to Australia in early 2015.

## The Study

The study was approved by the Queensland University of Technology (Brisbane, Queensland, Australia) (Human Research Ethics approval no. 1300000333). DENV-2 was isolated from a serum specimen by cultivation in *Aedes albopictus* mosquito C6-36 cells.

The virus was recognized by pan-flavivirus monoclonal antibodies 6B-6C1 (*8*) and 4G2 (*9*) and DENV-2–specific monoclonal antibodies 3H5 (*9*), 5H12, and 6B2 (*10*) in indirect immunofluorescence assays with infected C6-36 cells. However, it was not recognized by monoclonal antibody 6F3.1, which reacts with a serologic epitope ^9^RNTPFNMLKRE^19^ in the capsid protein of nonsylvatic strains of DENV-2.

The consensus sequence of the viral genome was obtained by using 3′ and 5′ random amplification of cDNA ends (*11**,**12*) and reverse transcription PCR of ≈1-kb overlapping regions of the genome. Sequences of purified cDNA fragments generated by reverse transcription PCR were determined by using the dye di-deoxy chain termination method at the Australian Genome Research Facility (Brisbane).

**Figure 1:**
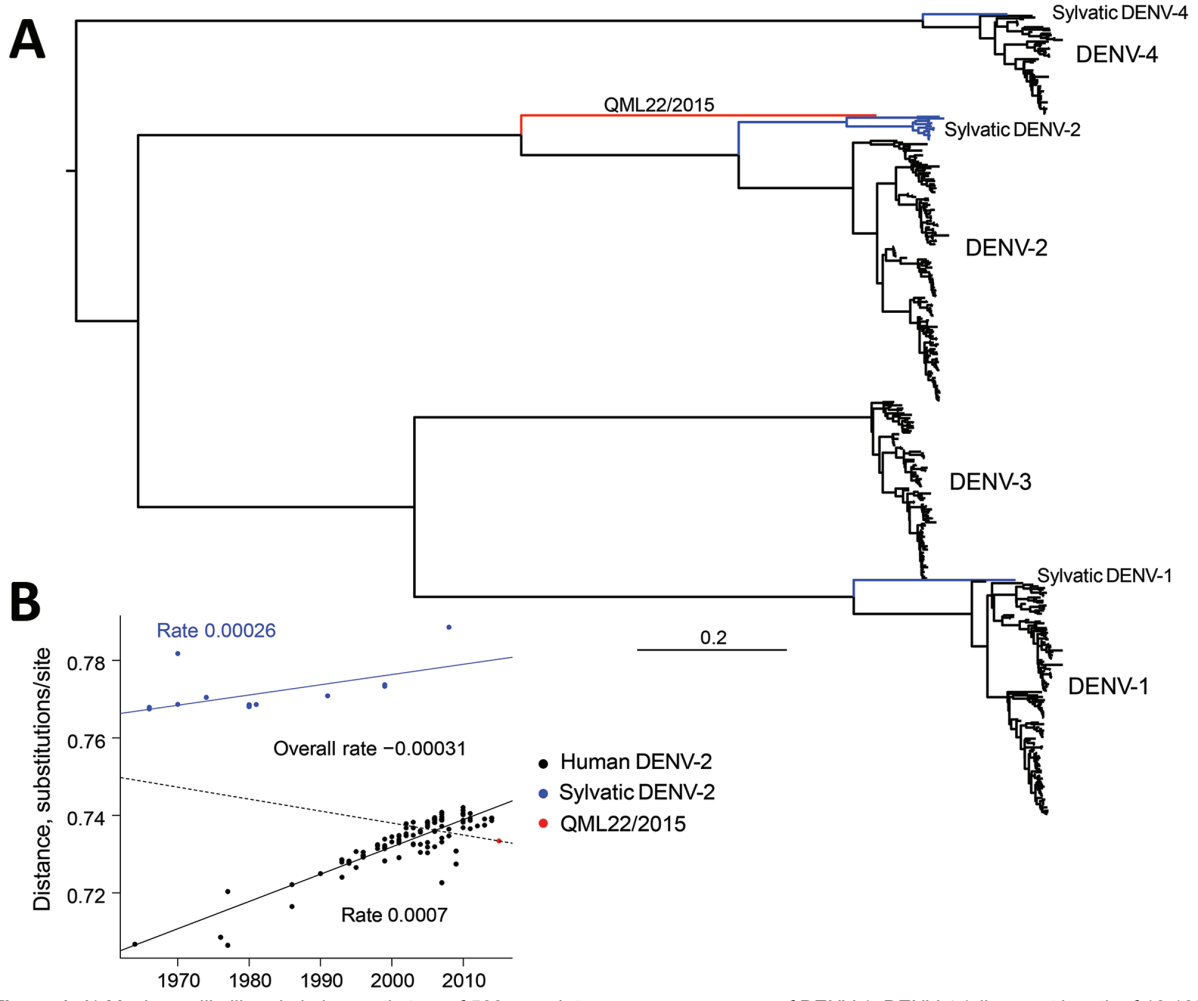
A) Maximum-likelihood phylogenetic tree of 500 complete genome sequences of DENV-1–DENV-4 (alignment length of 10,185 nt), including QML22/2015, estimated by using the generalized time-reversible invariable sites gamma model of nucleotide substitution in PhyML (*13*) and nearest-neighbor interchange plus subtree pruning, and regrafting branch-swapping. The tree is midpoint rooted for clarity, and sequences are color coded according to their putative transmission cycle (human, black; sylvatic, blue); red indicates the highly divergent QML22/2015 lineage isolated in this study. B) Regression of root-to-tip genetic distances of 119 representative human and sylvatic complete genome sequences of DENV-2 (alignment length 10,173 nt) against time (year) of sampling. The input phylogenetic tree was estimated by using the same maximum-likelihood procedure. Three regression lines and slopes are shown; slopes indicate an estimate of the virus nucleotide substitution rate (substitutions/site/year). Blue line indicates rate for entire DENV-2 data set; dashed line indicates rate for sylvatic DENV-2 sequences; black line indicates rate for human DENV-2 sequences. There is a marked difference between sylvatic and human rates. DENV, dengue virus.

Phylogenetic analysis of the complete viral genome (10,736 nt) by using maximum-likelihood methods (*13*) unambiguously placed this sequence, denoted QML22/2015 (GenBank accession no. KX274130), as a highly divergent member of DENV-2 with a strikingly basal phylogenetic position relative to all human and sylvatic DENV-2 sequences isolated (Figure 1). This lineage is the most divergent new lineage of DENV identified, even greater than that of DENV-1 Brun2014 (*7*), and is located approximately midway between the genetic divergence seen at the level of serotypes and that of genotypes within serotypes.

**Figure 2:**
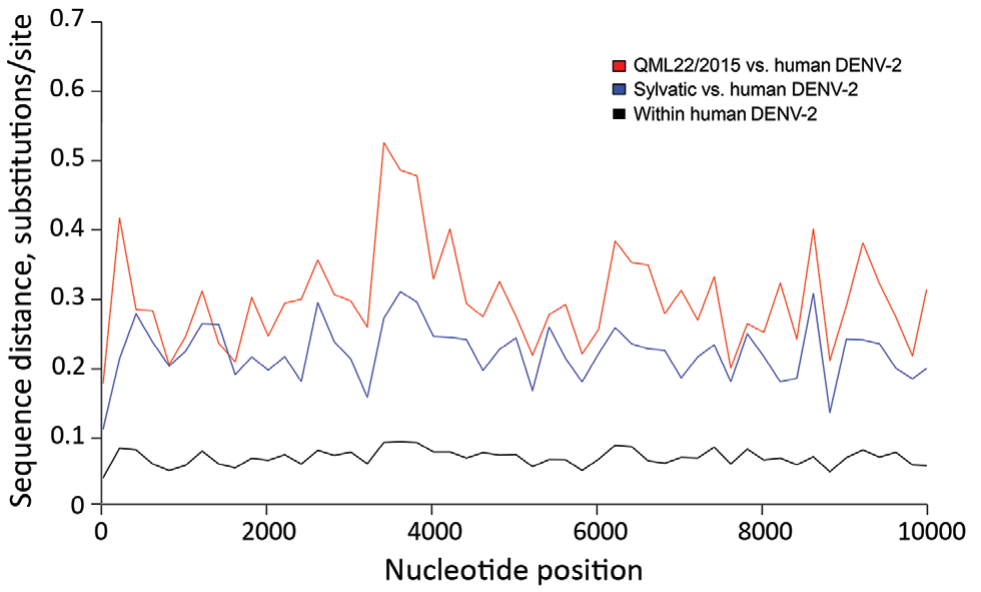
Sliding-window analysis of mean genetic (nucleotide) distance across the dengue virus type 2 (DENV-2) genome. Red line indicates comparison between QML22/2015 and human DENV-2 sequences. Equivalent analyses were performed on sylvatic DENV-2 versus human DENV-2 (blue line) and within the human DENV-2 sequences (black line). This analysis was based on genetic distances calculated by using sliding windows of 200 nt on the DENV-2 data described in Figure 1 and was performed by using the Analysis of Phylogenetics and Evolution Package in R software (*14*).

Although the nucleotide sequence of the open reading frame of QML22/2015 was strikingly different from those of other strains of DENV-2, sequence and predicted secondary RNA structure of 5′ and 3′ untranslated regions of the QML22/2015 genome were nearly identical with those of other strains of DENV-2, which confirmed the critical role of these elements in virus replication. Sliding-window analysis of genetic distance across the viral genome showed no regions in which QML22/2015 was disproportionately similar to human DENV-2 sequences (Figure 2).

## Conclusions

Although it is tempting to estimate the time of origin of this novel DENV-2, as performed for other divergent DENV lineages (*7*), we have not made this estimation because our data provided strong evidence for a marked difference in evolutionary rate between human and sylvatic strains of DENV-2, which will confound all attempts at molecular clock dating. Regression analysis of root-to-tip genetic distances against time (year) of sampling suggests that sylvatic strains of DENV-2 are evolving slower than urban (human) strains, most likely because of differences in selection pressure, replication dynamics, or both, and in contrast to previous observations (*15*).

The QML22/2015 isolate is closer to the human distribution than the sylvatic distribution of root-to-tip genetic distances, which suggests that this lineage might not have had only sylvatic transmission during its evolutionary history. Further studies are needed to determine whether this virus has infected other humans in Indonesia or other localities and identify genotypic changes that might give this virus distinctive phenotypic properties. The discovery of this and other highly divergent strains of DENV further emphasizes the need for biodiversity surveys of this major group of viruses in animals other than humans. It also suggests that gaps in DENV phylogeny might be filled after wider sampling.
